# An interseasonal outbreak of influenza A(H1N1)pdm09 related to a music festival, Denmark, August 2025

**DOI:** 10.2807/1560-7917.ES.2025.30.36.2500658

**Published:** 2025-09-11

**Authors:** Amanda Bolt Botnen, Hanne-Dorthe Emborg, Casper Thorup, Jesper Krog, Sofia Myrup Otero, Stine Nielsen, Bolette Søborg, Ramona Trebbien

**Affiliations:** 1 Department of Virology and Microbiological Preparedness, Statens Serum Institut, Copenhagen, Denmark; 2 Department of Infectious Disease Epidemiology and Prevention, Statens Serum Institut, Copenhagen, Denmark; 3 Department of Epidemiological Research, Statens Serum Institut, Copenhagen, Denmark

**Keywords:** influenza, integrated surveillance, outbreak, festival, gathering

## Abstract

In week 33, 2025, the integrated respiratory virus surveillance at Statens Serum Institut, Denmark, detected an atypical sixfold increase in influenza A(H1N1)pdm09 cases. Rapid sequencing of viruses collected in week 33 identified a highly related cluster of 17 cases. Telephone interviews with patients belonging to this cluster revealed that they all had attended a large open-air music festival. This is the first time an influenza outbreak has been detected during the summer in Denmark related to a large gathering.

The typical northern-hemisphere influenza season occurs between weeks 40 and week 20 the following year. Outbreaks outside of this timeframe are highly unusual. This rapid communication aims to investigate just such an outbreak during the summer of 2025 in Denmark. 

## Outbreak description

**Figure 1:**
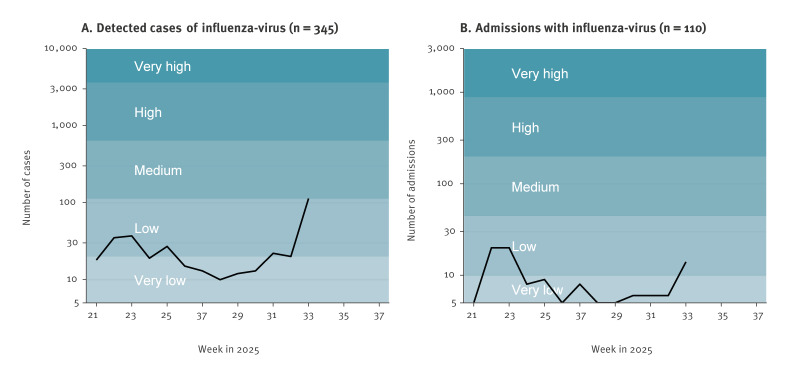
National summary of detected influenza cases and hospital admissions for influenza, Denmark, from week 21, 2025

On 19 August 2025, the integrated respiratory virus surveillance at Statens Serum Institut (SSI) detected a sixfold increase in the number of influenza A detections from 19 cases in week 32 to 115 in week 33, with only a slight increase in hospital admissions from six to 19 (Figure 1) [1]. There was an increase in the number of tests from 1,460 to 1,712 in weeks 32 and 33, respectively, although the average number of tests in the 2025 interseasonal period ranged from 1,412 to 2,207 tests per week. The cases were primarily from the Capital Region of Denmark and Central Jutland. The average age of cases in weeks 32 and 33 was 34 years and 21 years, respectively, with an age range of 3–83 years in both weeks. The average age during the preceding weeks 21 to 31 was 50 years (range: 0–91 years). The influenza season in Denmark is between weeks 40 and 20, as in many other countries in the temperate region of the northern Hemisphere. Outbreaks in the summer period are highly unusual and only reported occasionally [2,3].

Thresholds were generated using the aedseo R package, the method applied in Danish national surveillance to monitor changes in the circulation of respiratory infections [14].

## Laboratory analysis

**Figure 2:**
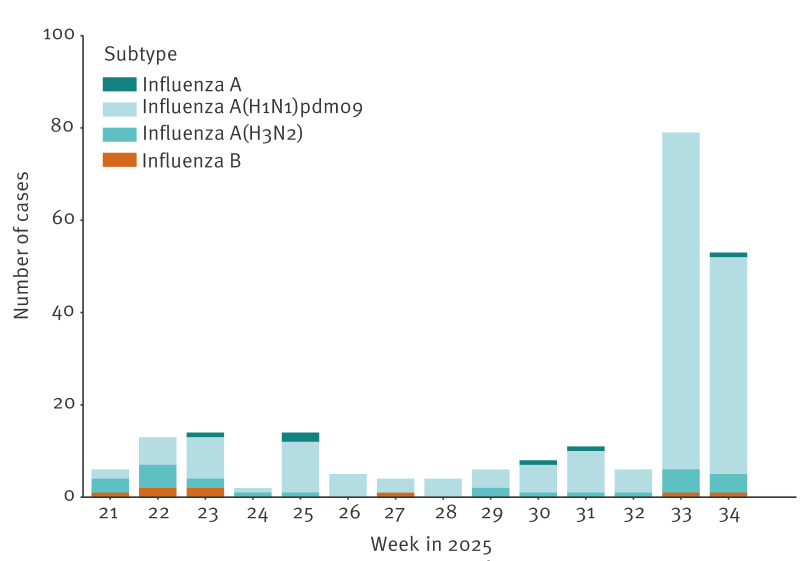
Subtype distribution of influenza A and B, received at Statens Serum Institut, Denmark, since week 21, 2025 (n = 225)

The influenza surveillance at the National Influenza Centre (NIC) at SSI receives respiratory samples from patients from two main sources: the primary care sentinel surveillance system and a subset of influenza-positive samples from regional clinical microbiological laboratories (CMLs). All sentinel submissions are routinely screened for nine respiratory viruses, including subtyping of influenza A viruses. In the interseasonal period , all CML submissions are subtyped. A total of 42 and 56 samples were received from the sentinel surveillance in weeks 32 and 33, of which 0 and 15, respectively, tested positive for influenza A(H1N1)pdm09. The CMLs submitted eight and 64 influenza-positive samples in weeks 32 and 33, respectively. Of these, influenza A(H1N1)pdm09 was detected in five samples from week 32 and in 57 samples from week 33 (Figure 2).

On 20 August, in week 34, we selected a subset consisting of eight sentinel and 13 CML samples for whole genome sequencing and initial characterisation. One sample was sequenced alone on Oxford Nanopore Technologies GridIon using the Rapid Sequencing Kit v14 for rapid characterisation. Another sample from the same patient was included in a sequencing run with the remaining subset of samples, using the ONT Rapid Barcoding Kit 24 v14. In total, samples from 20 unique patients were sequenced in this initial round.

All sequenced samples belonged to haemagglutinin (HA) clade 5a.2a.1 subclade D.3.1. Interestingly, viruses from 17 patients were nearly identical (nucleotide consensus identity 100% for most HA and all neuraminidase (NA) segments) and had two HA1 substitutions not previously detected in the Danish influenza surveillance. One substitution, HA1:S157L, was in the Sa antigenic site. The other substitution was HA1: V321I. The NA segments from these 17 patients all belonged to NA clade D.1 and had two substitutions, NA:M314I and NA:N341S. Both NA substitutions had previously been observed individually in the national surveillance, but never in combination. For the three viruses unrelated to the cluster, the NA substitutions were not present, and one of these three viruses was classified as belonging to NA clade D.2.

**Figure 3:**
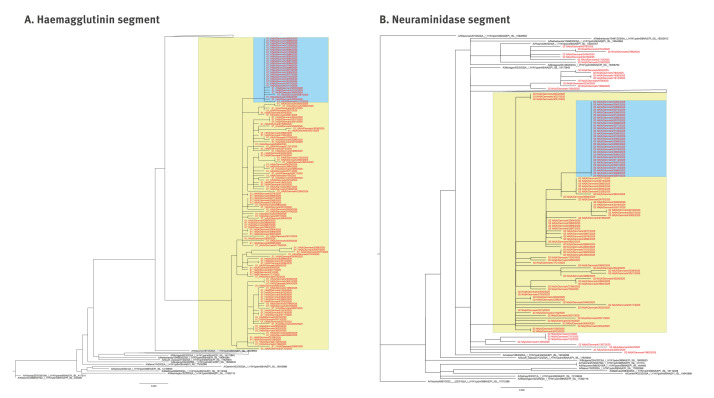
Phylogenetic tree of influenza H1 and N1 segments in the national influenza surveillance, Denmark, 2024/25 season (n = 29 outbreak sequences)

Subsequent sequencing (Illumina NextSeq 1000 with modified DNA prep kit) of 49 influenza A(H1N1)pdm09-positive samples from the national influenza surveillance from weeks 29 to 33, including the 21 previously Nanopore-sequenced samples and 28 new ones, revealed 11 additional patients with viruses similar to the identified outbreak virus. These 11 patients had sample collection dates between 11 and 14 August in week 33. Sequencing revealed 20 patients swabbed in week 27 to 33 with influenza A(H1N1)pdm09 infections not related to the outbreak. This brought the total number of patients with sequenced viruses belonging to the outbreak to 28 (Figure 3).

Viruses collected in the Danish surveillance are coloured with red labels.

The trees are based on the Illumina sequences. Blue box: outbreak viruses. Yellow box: subclade D.3.1 in panel A, subclade D.1 in panel B.

## Interviews

We initially prepared a line list comprising 20 influenza A(H1N1)pdm09-positive patients swabbed during the first 3 days of week 33 for whom whole genome sequencing data were available. Of these, three could be excluded from the event based on WGS results, one had left the country, and one had no available contact information. The remaining 15 individuals were contacted by telephone for a hypothesis-generating interview, of whom 10 completed the interview. All 10 had attended a music festival, Smukfest in Central Jutland [4], in week 32. The patients had spent the nights in different locations, and some stated that many other participants had fallen ill during and after the festival. None of them had participated in other large gatherings or travelled abroad, and one had been hospitalised in relation to this illness. We also interviewed two of the three patients with viruses distinct from the outbreak virus; neither had attended the music festival, and both had travelled abroad and returned to Denmark right before they tested positive. Only one of the 20 patients had been vaccinated against influenza in the 2024/25 season.

## Discussion

An atypical influenza A signal was first detected by the integrated respiratory virus surveillance at SSI in the late morning on 19 August 2025. By early afternoon the following day, the NIC had sequenced and identified a cluster of highly similar influenza A(H1N1)pdm09 belonging to HA clade 5a.2a.1 subclade D.3.1. This cluster had two HA substitutions not previously detected in the national seasonal surveillance. All interviewed patients identified as part of the outbreak had attended the same music festival in week 32. Approximately 60,000 visitors per day attend Smukfest’s week-long open-air music festival [4].

We speculate whether the A(H1N1)pdm09 outbreak virus has features that could explain the successful transmission; however, we identified only a few genetic changes from the 2024/25 seasonal vaccine strain A/Victoria/4897/2022 and circulating seasonal viruses in DK. One amino acid substitution was in the haemagglutinin protein at antigenic site Sa at position HA1:S157L. Further investigations are required to explore the phenotypic features of the outbreak virus.

Descriptions of super-spreading events [5] of influenza A(H1N1)pdm09 are not common but have been reported on a few occasions, e.g. in France in an outbreak connected to transportation by train [6]. Outbreaks in schools or nursing homes with a specific influenza subtype during the influenza seasons are common [2,7-9]. Super-spreading events were found to drive transmission of severe acute respiratory syndrome coronavirus 2 (SARS-CoV-2) during the COVID-19 pandemic, but this has not been the case for influenza A(H1N1)pdm09 [10]. Open-air festivals were linked to increased transmission of influenza A(H1N1)pdm09 in 2009 [11], and large gatherings with close contact and singing, such as concert settings, are considered a risk for transmission of respiratory viruses [12].

School outbreaks mainly affect the younger part of the population, and few severe cases have been reported [7]. However, outbreaks in nursing homes, with a more vulnerable population, often give rise to hospitalisation and deaths, seen in the case fatality rate of 15.6% in an outbreak in France [2]. Participants in open-air festivals will often be adults outside the usual risk groups for influenza (i.e. not children 0–5 years and people > 65 years). The average age of participants at Smukfest is 39 years [13] and as such, it is expected that the proportion of severe cases will be smaller than seen during a usual influenza season.

## Conclusion

The high identity of the virus sequences obtained from the music festival outbreak in Denmark suggests involvement of super-spreading from a single individual. The outbreak is highly unusual, as out-of-season outbreaks have only been observed in connection with pandemics in Denmark. This is the first time an influenza outbreak indicating a super-spreading event has been detected in the summer in Denmark.

## Data Availability

The individual-level data used in this study are sensitive and cannot be publicly shared. Sequences have been deposited in GISAID under EPI_ISL numbers 20139735–20139752, 20139754–20139760 and 20139763–20139766.
